# Utility of Germline, Somatic and ctDNA Testing in Adults With Cancer

**DOI:** 10.1002/cam4.71080

**Published:** 2025-08-01

**Authors:** Emily DeBortoli, Ella McGahan, Tatiane Yanes, Jennifer Berkman, Lauren G. Aoude, Amelia K. Smit, Akira Gokoolparsadh, Azure Hermes, Lyndsay Newett, Mackenzie Bourke, Susan Hanson, Helen Hughes, Oliver Hofmann, Ilias Goranitis, Rebekah McWhirter, Vivienne Milch, Julia Steinberg, Aideen McInerney‐Leo

**Affiliations:** ^1^ Dermatology Research Centre, The Frazer Institute The University of Queensland Brisbane Queensland Australia; ^2^ Frazer Institute The University of Queensland Brisbane QLD Australia; ^3^ The Daffodil Centre The University of Sydney, a Joint Venture With Cancer Council New South Wales Sydney New South Wales Australia; ^4^ Faculty of Medicine and Health, Sydney School of Public Health The University of Sydney Sydney New South Wales Australia; ^5^ Faculty of Medicine and Health, Sydney School of Public Health, Sydney Health Ethics The University of Sydney Sydney New South Wales Australia; ^6^ National Centre for Indigenous Genomics Australian National University Canberra Australia Capital Territory Australia; ^7^ Economics of Genomics and Precision Medicine Unit, Centre for Health Policy, Melbourne School of Population and Global Health University of Melbourne Melbourne Victoria Australia; ^8^ Cancer Australia Sydney New South Wales Australia; ^9^ University of Melbourne Centre for Cancer Research Melbourne Victoria Australia; ^10^ Australian Genomics, Murdoch Children's Research Institute Melbourne Victoria Australia; ^11^ School of Law Australian National University Canberra Australia; ^12^ Caring Futures Institute Flinders University Adelaide South Australia Australia

**Keywords:** cancer, ctDNA, genetics, genomics, germline, narrative review, somatic

## Abstract

**Background and Aim:**

Historical genetic sequencing of specific cancer variants has been superseded by comprehensive genomic profiling (CGP). This narrative review aimed to capture current international evidence on the clinical utility of CGP for cancer prevention, detection and treatment.

**Materials and Methods:**

A literature search of three databases was performed to identify key studies on the frequency of germline and somatic variants in adult cancers and the extent to which they inform diagnosis, management and outcome. Findings were inductively mapped and narratively synthesised.

**Results:**

Consolidated results from 95 original research papers showed that pathogenic germline (familial) variants are found in ~10% of adults with cancer, of whom 53%–61% are offered germline genotype‐directed treatment. Importantly, 50% of germline carriers would not have satisfied the eligibility criteria for genetic testing and/or reported a negative family history. Actionable somatic variants occur in 27%–88% of cases, which markedly impact the diagnosis for cancers of unknown primary. Matched treatments were identified for 31%–48% of cancer patients, of whom 33%–45% received it. Response and survival rates were better in individuals receiving matched therapies compared to those receiving standard of care or unmatched therapies. Trials show that circulating tumour DNA (ctDNA) assays are feasible and sensitive. The relatively non‐invasive ctDNA sample collection is appealing for cancers with inaccessible or unknown primary sites, and serial monitoring of residual disease and/or treatment response.

**Conclusions:**

As matched therapies are underutilised due to declining patient condition and fewer prior therapies predicting better response rates, research is needed on the suitability of cancer genomic profiling as a frontline test.

## Graphical abstract

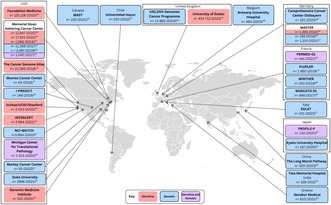

## 1 Introduction

Genomic variants can contribute to cancer susceptibility and development, as well as treatment responsiveness and resistance. Prior to the last decade, hereditary cancer genomic testing comprised time‐consuming, sequential sequencing of high‐penetrance genes in cancer families to determine susceptibility, while precision genomic testing was limited to a handful of variants within specific cancers that could guide management (e.g. *BRAF* V600E) [1]. Following the introduction of next‐generation sequencing (NGS), hereditary cancer susceptibility and tumour profiling typically utilise large panel sequencing (hundreds of genes), exome sequencing (coding region of all ~20,000 genes) or whole genome sequencing (WGS) (coding and non‐coding regions of the genome). Exome and WGS data can be interrogated agnostically, or more commonly, filtered for variants in genes involved in cancer predisposition or pathogenesis (virtual panels) [2]. Panels and exomes generate a more manageable quantity of data and interpretation workload relative to WGS, but are comparatively less sensitive in detecting copy number variants (CNVs) or chromosomal rearrangements [2, 3].

Rare germline variants are associated with hereditary cancer syndromes, typically characterised by an early age of onset, two or more primary cancers in an individual and an autosomal dominant family history of related cancers [4]. Common syndromes include hereditary breast and ovarian cancer, Lynch syndrome, Li‐Fraumeni syndrome, multiple endocrine neoplasia, neurofibromatosis and hereditary phaeochromocytoma‐paraganglioma syndromes [5]. Associated high‐risk genes, including *BRCA1/2*, *TP53, MEN1, NF1, MLH1, MSH2, APC, RET* and *VHL*, are known to significantly increase cancer risk [6]. Typically, germline genetic testing for hereditary cancer syndromes is based on personal and family history criteria and is only offered when the a priori risk is ≥ 10% [7, 8]. Germline variants in hereditary cancer genes are classified on a continuum according to the likelihood of causality, from pathogenic, likely pathogenic, variant of uncertain significance, likely benign, to benign [9].

Somatic genetic variants arise in cells throughout life, either due to exposure to mutagens/carcinogens or through cellular replication and division [10]. Tumour somatic genomic profiling aims to identify actionable variants that inform diagnosis, prognosis and/or guide treatment selection and management [11]. The American Society of Clinical Oncology and College of American Pathologists classify somatic pathogenic/likely pathogenic variants (P/LPVs) into four tiers in decreasing order of clinical significance, where Tier I and Tier II are most frequently actioned [12]. Of note, multiple other classification systems exist for somatic variant actionability, including the European Society for Medical Oncology's Scale of Clinical Actionability of molecular Targets (ESCAT) [13]. The frequency of variants within a tumour sample can be used to estimate tumour purity, though this is modified by tumour type, heterogeneity and the nature of somatic changes (e.g. single nucleotide polymorphisms [SNPs], small insertions or deletions of nucleotide bases [indels], CNVs) [14].

Upon cellular death, cell‐free DNA (cfDNA) is released into the bloodstream, and in cancer patients, a portion of this is comprised of circulating tumour (ctDNA). Extraction of ctDNA from blood, urine and cerebrospinal fluid, otherwise known as a liquid biopsy, is much less invasive than a tissue biopsy [15]. P/LPVs can be detected through ctDNA sequencing (NGS or digital polymerase chain reaction [PCR]) and the most widely used application of ctDNA is the monitoring of minimal residual disease or response to treatment using a tumour‐informed assay (tracking patient‐specific mutations) or a tumour‐agnostic approach (a panel of common, predefined signatures) [16].

Testing and treatment terminology pertaining to cancer genomic profiling is constantly evolving. Table 1 contains definitions and examples of genetic aberration patterns (e.g. tumour mutational burden (TMB), homologous repair defect (HRD) scores, microsatellite instability (MSI) and aberrant methylation) and therapies (e.g. targeted therapies, small molecule inhibitors and monoclonal antibodies). Of note, of the oncology drugs approved since 1998, 83% are targeted therapies [23, 24], and over half of these are precision oncology therapies (drugs with maximal efficacy in a molecularly defined subset of patients) [25].

**TABLE 1:** Definitions commonly utilised in cancer genomic testing.

Term	Description
Cancer mutational signatures	Characteristic patterns of genetic variants found throughout the genomes of cancer cells, arising from disruptions in DNA repair processes
Copy number variants (CNVs)	Sections of the genome are repeated or deleted (> 1000 bases in size)
Circulating tumour DNA (ctDNA)	DNA shed by tumour cells into the bloodstream
Driver variants	Variants/mutations which drive the development, growth and invasion of cancer cells
Gene fusion	Genomic rearrangements leading to the fusion of two genes and subsequently abnormal protein production
Homologous repair defect (HRD) scores	HRD score is an unweighted sum of three independent DNA‐based measures of genomic instability (loss of heterozygosity, telomeric allelic imbalance and large‐scale transitions) [17] A High HRD score is often classified as ≥ 42 can arise due to germline and/or somatic variants [18]
Liquid biopsy	A genetic sample extracted from blood that can include ctDNA, circulating tumour cells, protein biomarkers and cell‐free RNA
Matched therapy	Selection of a drug (clinically approved or in clinical trial) based on evidence of efficacy in that cancer or another cancer with the same molecular profile
Matched tumour‐normal samples	Both tumour and unaffected samples (e.g. blood or saliva) are collected and tested simultaneously to determine whether variants are germline or somatic
Methylation defects	Methylation is a form of epigenetic modification, which enhances or suppresses gene expression Hypomethylation (overexpression) is widespread in cancer genomes Hypermethylated (suppression) often occurs in tumour suppressor genes Cumulatively, aberrant methylation contributes to genomic instability [19]
Microsatellite instability	Microsatellites are short sets of repeated DNA that are not present in the corresponding germline DNA. High levels of microsatellite instability (expansion of ≥ 30% microsatellite sites) [20] can contribute to genetic instability in cells Associated with, but not pathognomonic for Lynch syndrome [21]
Monoclonal antibodies (mABs)	Monoclonal antibodies block the binding of molecules that cancer cells need to grow, flag cells for destruction by the immune system or facilitate intracellular delivery of drugs, toxins or radioactive particles [22]
Polygenic risk score (PRS)	A numerical assessment to summarise an individual's genetic susceptibility to a particular trait or disease (such as cancer), based on many genetic markers across the genome
Small molecule inhibitors	Small molecule inhibitors target proteins both within the tumour cell and on the surface, for example, tyrosine kinase inhibitors (TKIs) and mammalian target of rapamycin (mTOR) inhibitors [22]
Targeted therapies	Targeted therapies block molecular pathways that are key for cancer cell growth and metastasis. Most biomarker specific therapies can be grouped into two categories: small molecule inhibitors and monoclonal antibodies
Tumour mutation burden (TMB)	The number (or rate) of somatic variants in the DNA of cancer cells. A higher TMB is commonly defined as ≥ 10 mutations/megabase
Variant of uncertain significance (VUS)	A genetic variant whose role in disease is not yet understood or determined

Recently, the applications and utilisation of cancer genomic testing have expanded exponentially [11]. This review aimed to capture how genomic sequencing (germline and somatic) is utilised in cancer patients and those at increased risk for developing cancer. Specifically, we will determine the frequency of germline and somatic P/LPV variants, and the extent to which they inform risk, diagnosis, prognosis and treatment. The review will also summarise the evidence regarding the applications and potential utility of ctDNA in adult cancers.

## 2 Methods

We undertook a narrative review of the current applications of genomic profiling in adult cancers. A literature search of PubMed, Google Scholar and Web of Science was last performed in January 2024 using key search terms: cancer, genomic profiling, germline, somatic and specific keywords (e.g. ctDNA, MSI, TMB, HRD and methylation defects) (Table S1). Prominent papers published since the initial search have also been added. The references and citations for all included articles were reviewed to identify other large eligible studies, and title/abstract and full‐text screening of articles were performed by A.M.‐L., E.D. and E.M. The search was limited to studies published in English from 2017 onwards, though a small number of key large studies published between 2012 and 2017, which were referenced by selected papers, were included. Single cancer type and/or small cohort size (*n ≤* 100 patients) papers were omitted to ensure the representation of general cancer cohorts. The key findings of eligible articles were inductively mapped and narratively synthesised.

## 3 Results

**FIGURE 1:**
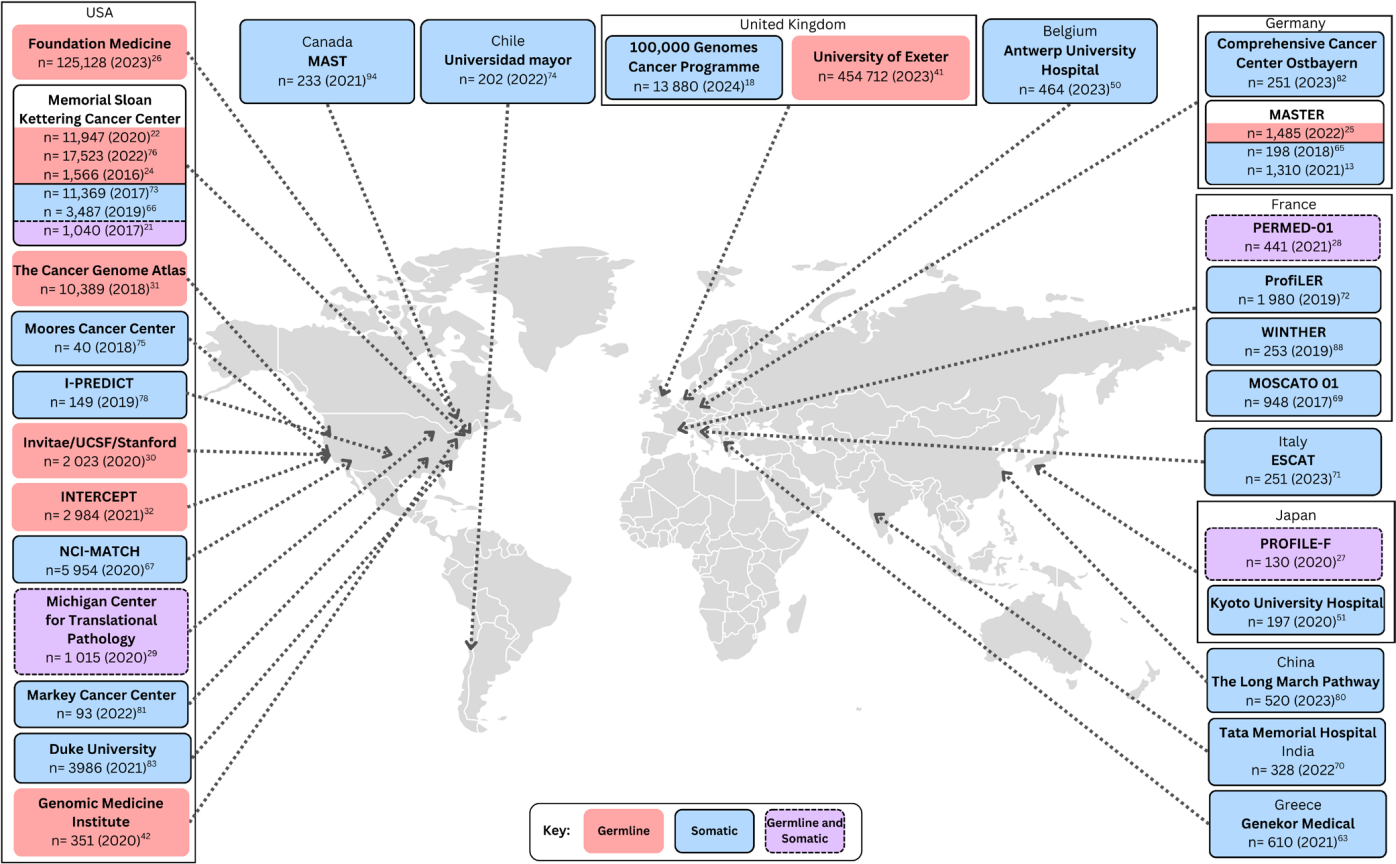
Large multi‐cancer studies internationally that included germline and/or somatic genomic profiling.

**FIGURE 2:**
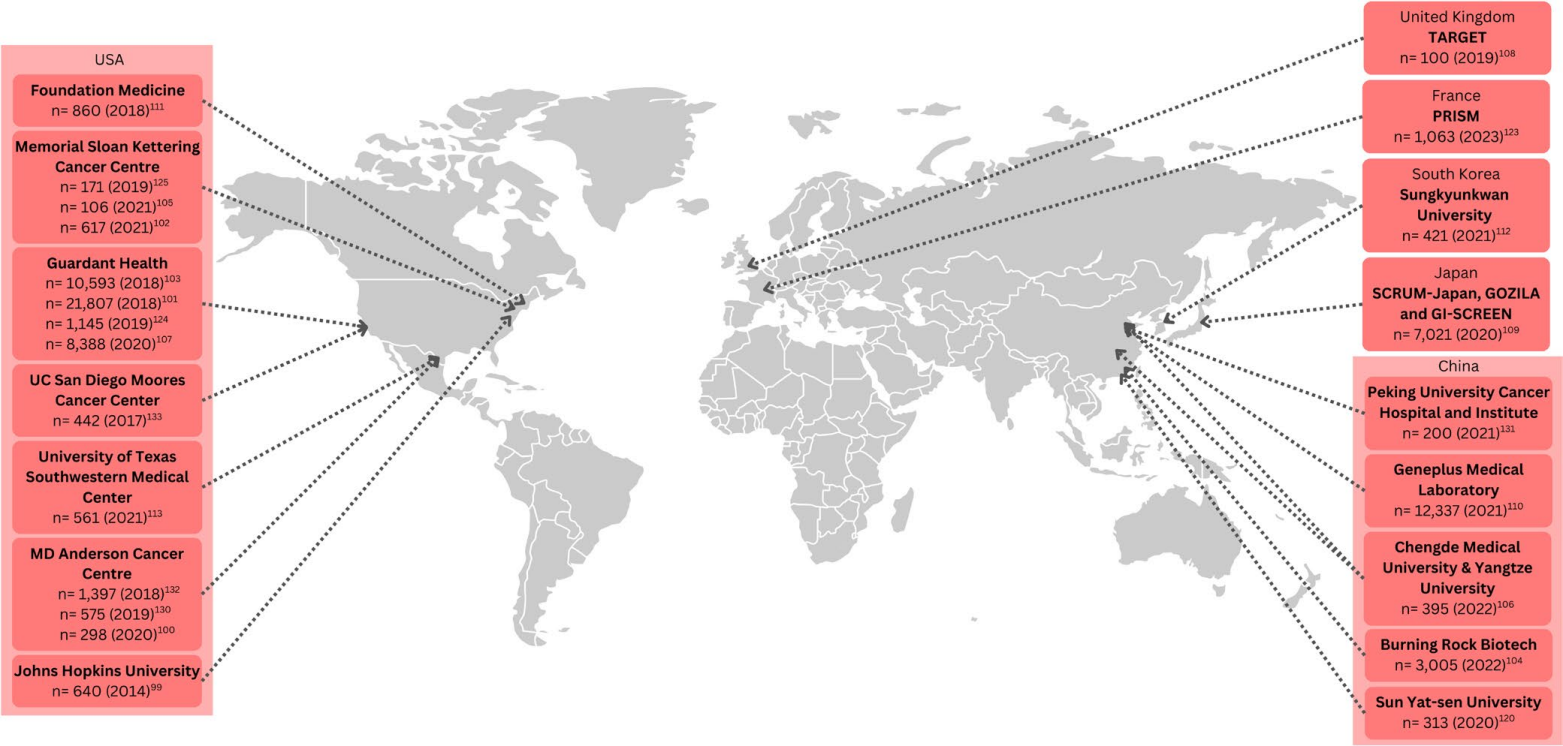
Studies internationally that included ctDNA analysis in primarily advanced cancer cohorts.

The titles/abstracts of 1312 articles were initially screened for eligibility, and 126 underwent full‐text review. Of the 120 articles included in this review, 95 (79%) were original articles, 20 (17%) were reviews and 5 (4%) were relevant guidelines. Most (51%) original articles originated from the United States of America (USA) and the remainder were largely from Europe (20%), Asia (19%) and Australia (8%). Overall, 78 articles (68%) pertained to germline/somatic profiling, and 37 (32%) to ctDNA, excluding guidelines. Figure 1 summarises the location and sizes of larger germline and somatic cancer genomic profiling studies internationally, which included populations with diverse cancer types (i.e., studies on single cancer types were not included in this map). Of note, 204,487 samples had somatic and/or germline analysis in the USA and 478,393 in other countries combined. Figure 2 captures the location and scale of ctDNA studies for diverse cancer types (predominantly in advanced cancer) internationally, where two‐thirds of ctDNA samples analysed originated from the USA (*n* = 47,600) and the remaining third (*n =* 24,855) from other countries worldwide. Table 2 synthesises the relevance of genomics for adult cancers relative to the cancer care continuum, from prevention and early detection to palliative care and end of life [71].

**TABLE 2:** Potential utility of genomic testing relative to the cancer care continuum for adults with cancer.

Stage	Variant	Adult cancers
Prevention and early detection	Germline	Predictive testing for familial cancers allows for personalised screening, surveillance and prophylactic interventions that mitigate risk and result in early detection and improved outcomes [26, 27, 28, 29] PRS can aid in further risk stratification in germline carriers [30, 31, 32]
	Somatic	N/A
Presentation, initial investigation and referral	Germline	N/A
	Somatic	ctDNA analysis has been trialled for individuals presenting with possible cancer symptoms in general practice, however there are feasibility, sensitivity and clinical utility challenges [15, 33]
Diagnosis, staging, planning	Germline	~10% of adults with cancer carry a germline P/LPV [34, 35] Higher P/LPV detection rates are reported in rare cancers (13%–18%) [36, 37, 38] and using larger panels [39] Germline P/LPV frequencies are higher in certain cancers (see Figure 1) 50% of adults found to carry germline P/LPVs would not have met eligibility criteria [36]
	Somatic	27%–88% of tumour samples have a P/LPV which inform diagnosis, prognosis and/or treatment selection [13, 38, 40, 41, 42, 43, 44, 45, 46, 47, 48, 49, 50] which increases to 88%–93% for rare cancers P/LPV [13, 51] 85% of somatic P/LPV are single nucleotide variants or indels, 12% are copy number variants and 3% are gene fusions [52] 4%–10% advanced cancer patients [13, 53] and 51% of CUP patients [38] have P/LPV informing/refining diagnosis
Treatments, clinical trials and outcomes	Germline	53%–61% of individuals with somatic P/LPV were offered germline genotype‐directed therapies [39, 54]
	Somatic	Matched therapies were identified for 31%–48% of patients, of whom 33%–45% received it [13, 45, 46, 47, 53, 55, 56] Partial/complete/overall response rate to matched therapy was between 11% and 52% [57] PFS [44, 49] and median OS [44, 49] was higher in advanced cancers receiving matched therapy Tier I or Tier II therapies had better PFS and OS than those with lower evidence levels [49, 56, 58] Individuals receiving matched therapies who had fewer prior therapies showed better PFS and OS [46, 59] Serial TMB can be used to detect response to therapy [60]
Care after initial treatment	Germline	Adjuvant therapies can mitigate the risk of metastases for some germline P/LPV carriers [61] Inform future cancer risks and facilitate cascade testing amongst families of germline P/LPV carriers [62, 63] Custom screening and interventions can prevent second primary or facilitate earlier detection [64]
	Somatic	Large research studies demonstrate that ctDNA analysis can be applied to predict recurrences [65, 66] Increasing studies exploring the use of methylation as a biomarker, especially in breast cancer [67]
Managing refractory, relapsed or progressive disease	Germline	PRS can refine risk for a subsequent cancer [68, 69, 70]
	Somatic	38% of patients with refractory cancer had an actionable somatic variant and half were assigned to a treatment protocol [45] Pancreatic cancer patients with a poor prognosis who received matched therapies have longer median OS and longer OS compared to those who received unmatched therapies [55]
Palliative care and end of life	Germline	N/A
	Somatic	N/A

Abbreviations: CUP, cancer of unknown primary; OS, overall survival; P/LPV, pathogenic or likely pathogenic variant; PFS, progression‐free survival; PRS, Polygenic Risk Scores; TMB, tumour mutational burden.

### 3.1 Germline Genomic Variants in Adult Cancers—Familial Variants

**FIGURE 3:**
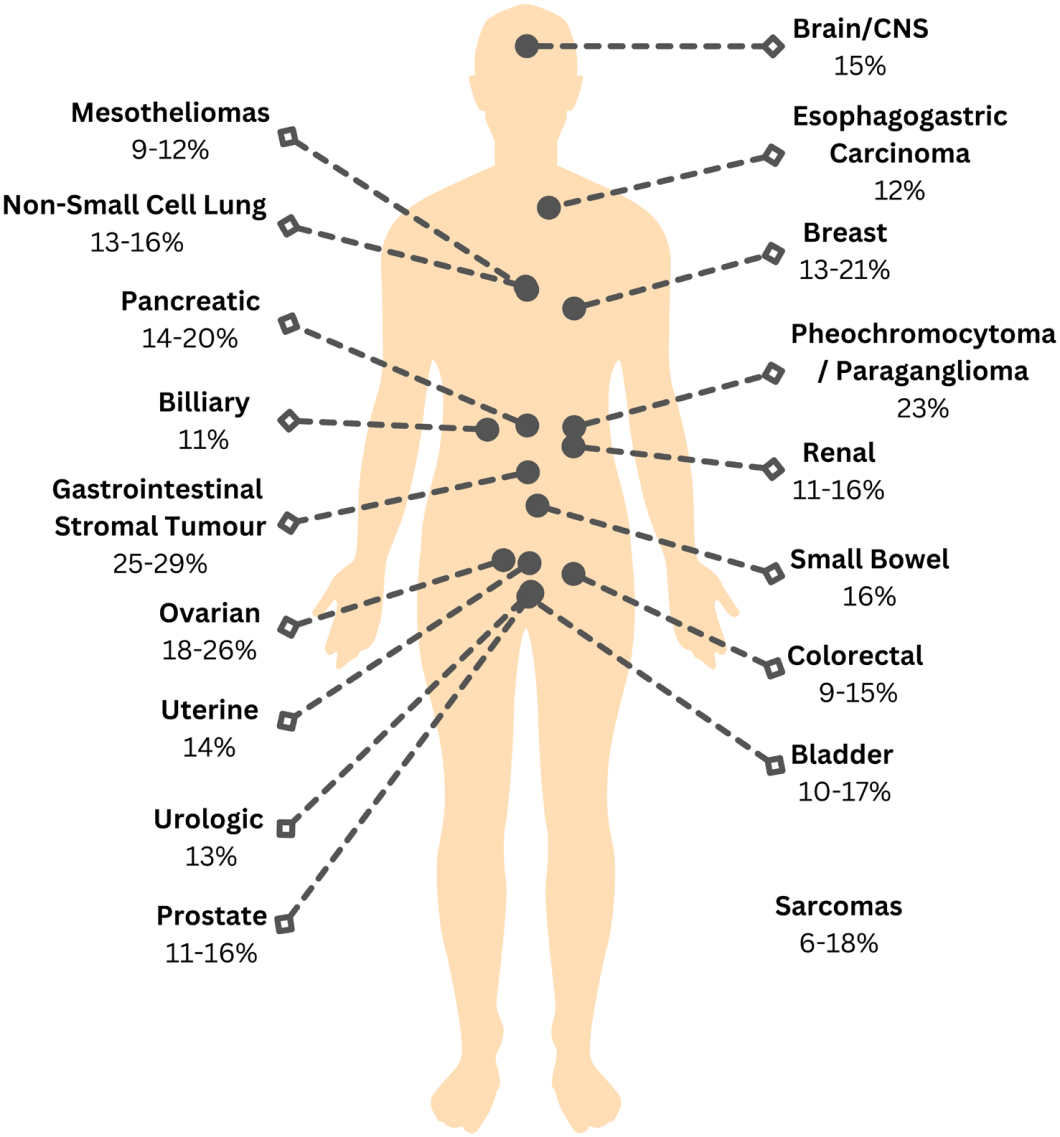
Adult cancers associated with the highest frequency of germline variants.

Germline P/LPVs are currently identified in 9%–17% of adults with cancer [13, 34, 35, 36, 37, 38, 39, 40, 41, 54], with two recent, large studies identifying germline P/LPV in 10.5%–10.6% [34, 35]. Higher germline P/LPV detection rates are reported in rare cancers (13%–18%) [36, 37, 38], and cohorts enriched for positive family history (30.5%) [64]. Cancers most likely to harbour germline P/LPV are summarised in Figure 3 [34, 39, 54, 72]. Data from four large cohorts were used to create this figure, but there are diverse ranges depending on whether variants of all penetrance types (high, moderate and low) and/or gene inheritance type (autosomal dominant and recessive) were reported. One study reported variants of all penetrance levels [39], another provided two types of penetrance results (one for moderate‐high penetrance and one for all penetrance types) [34], while the remaining two did not comment on the penetrance of the reported variants [54, 72]. Additionally, three of these studies reported P/LPVs cumulatively, regardless of whether they occurred in dominant or recessive genes [39, 54, 72].

Three‐quarters of P/LPV carriers identified through cancer genomic profiling were unaware of their germline status [54], and > 50% of cancer patients with clinically actionable germline P/LPVs would not have met eligibility criteria for germline testing [36, 73]. Notably, in one large study (*n =* 12,176) conducted between 2021 and 2022, 64% of germline P/LPV carriers presented with cancer types that lacked explicit hereditary cancer testing guidelines [35]. Reflex germline testing for relevant genes is recommended for epithelial ovarian cancer, exocrine pancreatic cancer and metastatic prostate cancer due to the recognised benefits of DNA repair targeted therapies [74, 75, 76, 77] and in those with colorectal cancer meeting immunohistochemistry criteria [78], due to improved outcomes with immune checkpoint inhibitors [79].

### 3.2 Germline Variants in Asymptomatic Population—Familial Variants

Genetic testing for high‐risk germline P/LPVs in asymptomatic individuals has been trialled in general populations to inform cancer prevention and early detection strategies. Approximately 0.5% of North American biobank cases (*n* = 267/50,459) carried a *BRCA1/2* P/LPV [80]. Similarly, 0.64% of healthy Australian women (*n =* 38/5908) had a P/LPV in 1 of 11 high‐risk breast/ovarian cancer genes [81]. In the UK Biobank, 0.33% (*n* = 841/256,591) of women with a pathogenic variant in *BRCA1/2*, 70% reported no first‐degree relatives with the associated cancers, indicating that testing based on family history criteria alone fails to detect a substantial number of carriers [82]. Genetic testing in an unselected USA clinical population for hereditary breast and ovarian cancer and Lynch syndrome found that 82% (*n* = 305/351) of P/LPV carriers had no prior genetic diagnosis, and 50% of these individuals reported a negative personal and family history (first and second‐degree relatives) [83].

### 3.3 Utility of Identifying Germline Variants in Cancer Susceptibility Genes

In an unselected USA clinical population, over 350 individuals were found to carry P/LPV for hereditary breast and ovarian cancer or Lynch syndrome, of whom 255 (73%) were eligible for risk management, and the majority (179/255, 70%) adhered to the recommended procedure [83]. In families with hereditary cancers, cascade testing (i.e., predictive testing in unaffected blood relatives) allows for tailored screening, preventative prophylactic surgery and chemoprevention for those who also carry the high‐risk P/LPV, facilitating early detection and better clinical outcomes [26, 27, 28, 29]. Of individuals with cancer known/found to carry germline P/LPV, between 53% and 61% were offered germline genotype‐directed therapies, many of whom were *BRCA1/2* positive [39, 54]. PARP inhibitors in *BRCA1/2* positive cases can improve progression‐free survival (PFS) and overall survival (OS) in breast cancer, ovarian cancer and pancreatic cancer [84, 85]. Additionally, individuals with colorectal cancer secondary to germline P/LPV in Lynch syndrome genes, receiving immune checkpoint inhibitor treatment, have an objective response rate of 46%–71% [86].

### 3.4 Somatic Genomic Variants in Adult Cancers ‐ Testing Feasibility and Uptake

DNA extraction and genomic/panel sequencing were successful for approximately 80%–90% of samples, regardless of whether the sample was fresh‐frozen or formalin‐fixed paraffin‐embedded [38, 41, 87]. Initially, in studies conducted between 2017 and 2020, tumour molecular profiling turnaround times were reported to vary from 19 to 21 weeks [13, 42], but more recent studies report a mean turnaround time of 5.5–7.5 weeks from sample collection to return of results [88]. The most comprehensive detection of actionable variants was achieved using WGS or WES with RNA‐sequencing (RNA‐seq) [13, 38]. The addition of RNA‐seq improved the detection of gene fusions and indels, the verification of intratumoral expression of single‐nucleotide variants (SNVs), evaluation of transcriptional effects of gene amplifications and deletions and diagnostic classification of unclear disease patterns [13]. In research settings, RNA‐seq also increased the detection of biomarkers [13], such as miRNA, lncRNA and circRNA, which could potentially be used to monitor disease progression and response to treatment [89, 90, 91]. Methylation studies are particularly valuable in the classification of brain tumours and sarcomas [92], and can enhance the identification of therapeutic targets and better predict response to treatment in breast cancer [67, 93].

Although beyond the scope of this review, it is noted that within most studies, patients and clinicians held positive attitudes regarding tumour molecular profiling [94, 95, 96]. Patients' uptake of testing was high (89%), though there was some hesitation expressed around willingness to pay for tests that are not fully publicly funded [97].

### 3.5 Somatic Variant Types and Frequencies

Somatic P/LPVs found in adult cancers include indels, large duplications or deletions (i.e., CNVs), gene rearrangements and fusion genes. Most (85%) somatic P/LPVs in adult cancers are SNVs or indels, 12% are CNVs, and 3% are gene fusions [52]. CNVs are detected in diverse cancer types, particularly sarcomas and uterine, oesophageal, ovarian and bladder urothelial carcinomas [98].

Most large studies to date have focused on advanced and/or rare cancers. Actionable somatic P/LPVs, which inform diagnosis, prognosis or treatment selection, are identified in 27%–88% of tumour samples, with most studies reporting an actionable variant in 73%–80% [13, 24, 38, 40, 41, 42, 43, 44, 45, 46, 47, 48, 49, 50]. The genes most likely to harbour actionable somatic P/LPVs include *PIK3CA, KRAS, PTEN, TP53, ERBB2, BRCA1/2, NRAS, PR, ER, BRAF, EGFR, AKT1, RET* and *cMET* [41, 43, 44, 45, 47, 49, 99, 100]. Patients with non‐small cell lung cancer (NSCLC), oesophageal cancer, ovarian cancer and cancers of unknown primary (CUP) were more likely to harbour actionable somatic variants [40]. Similarly, 88%–93% of rare cancers had ≥ 1 actionable somatic variant [13, 51]. A study of almost 5900 patients with refractory cancers found that 71% had resistance‐conferring genetic variants and 38% carried an actionable somatic variant [45]. Refractory cancers that are most likely to be assigned to a matched therapy include cholangiopancreaticobiliary cancers, melanoma, prostate cancer, uterine cancer and gastroesophageal cancer [45].

### 3.6 Utility of Somatic Variants

Cancer genomic sequencing informed or refined diagnosis in 4.4%–10.5% of advanced cancer patients [13, 53], and was particularly valuable in CUP [13], where it amended/facilitated diagnosis in 51% [38]. Overall, 31%–48% of adults had ≥ 1 molecular variant with a corresponding matched therapy [13, 42, 43, 44, 45, 46, 47, 48, 50, 51, 52, 53, 55, 58, 101, 102, 103, 104], most of which were Tier II or Tier III [38]. When restricted to Tier I and Tier II variants, 17%–56% of patients had a targetable variant [49, 52, 58, 100, 105]. Cancers most likely to harbour Tier I targetable variants include NSCLC, breast cancer, melanoma and colorectal cancer [45, 58]. Cancers with Tier II variants included bladder cancer, breast cancer, NSCLC, pancreatic cancer and sarcoma [45, 58], and up to 76% of recurrent/metastatic cancer patients with Tier II variants received PI3K‐Akt–mTOR therapies through clinical trials [50]. Therapeutic variants are more common in colorectal cancer, gastric cancer and pancreatic cancer (63%) [42]. The presence of patterns of aberrations, such as high TMB [106], MSI [79] and burden of CNVs [107], was associated with a positive response to immunotherapy [108, 109].

On average, 33%–45% (range 6%–62%) of individuals with a targetable variant received matched therapies, with most accessed through clinical trials [13, 38, 40, 41, 43, 44, 45, 46, 47, 48, 49, 50, 52, 59, 87, 99, 100, 101, 104]. The majority (91%) of individuals with CUP had ≥ 1 actionable variant, triggering a change in therapy recommendation for 64% of these patients [110]. Of the 41% of CUP cases with a matched therapy, 16% received it [110]. Within clinical trials, the main reasons for not receiving matched therapy were reluctance to change current treatment, patient preference, unsuccessful prior utilisation of matched therapy, limited access to matched therapy and deterioration of patient condition [13, 40, 41, 47, 101, 110]. In studies which offered profiling outside of a clinical trial, the main reasons for not utilising the recommended therapy included deteriorating patient condition, patient preference, limited access to targeted treatment, targeted therapy used currently or previously and the prohibitive cost of treatment [43, 48, 87].

### 3.7 Impact of Matched Therapies on Response and Survival

We identified 16 original research studies that reported treatment response and outcomes in tumour‐agnostic basket trials [58, 111], N‐of‐1 platform trials [46, 51], clinical trials [44, 56] and real‐world prospective or retrospective studies using molecular tumour boards or clinical advisory boards [13]. Most studies focused on patients with advanced cancers generally and intractable cancers specifically. Response rate studies reported the proportion of patients receiving matched therapies who had stable disease (58%) [43], partial response (38%) [111] or complete response (17%) [111], while most grouped partial/complete/overall response rate (11%–52%) [13, 44, 46, 58] or reported disease control rates after 6–34 months (55%) [13]. Control rates were significantly higher in those receiving a matched therapy as compared to the 5%–30% of the remaining advanced disease cohort [43, 44, 46, 49].

Median PFS was 1.5‐fold higher in matched versus unmatched therapy groups [44, 49]. Median increase in OS ranged from 1.2‐fold (8.4 versus 7.3 months) [44] to 4.1‐fold (35.1 versus 8.5 months) [49]. Patients with matched therapies had significantly longer OS (16.9 versus 10.4 months) [56] than those with unmatched therapies or PFS (19.7 versus 3.5 months) relative to prior unmatched treatment [51]. A USA centre that reflexively performed genomic profiling in all advanced cancer patients found that individuals receiving genomically informed therapies had better OS at 12 months compared to patients receiving chemotherapy (70% versus 63%) [112]. Individuals receiving a matched therapy had a PFS2/PFS1 ratio (ratio of PFS time post current treatment as compared to PFS following prior treatment) > 1.3 in multiple studies [13, 41, 47, 49, 113]. Furthermore, those receiving Tier I [49] or Tier I/II therapies [58] had better PFS and OS than patients receiving therapies with lower evidence levels. Similarly, patients who had a ‘strong match’ according to molecular tumour boards had a longer PFS [59, 101] and OS [56, 101] than those with a ‘low match’ score, and PFS and OS were significantly higher in genomically informed therapy groups as compared to those receiving physician choice regimes [46]. Individuals with cancers associated with a poor prognosis (e.g. pancreatic) receiving matched therapies had longer median OS (2.58 versus 1.51 years) and a longer OS (2.58 versus 1.32 years), compared to those who received unmatched therapies [55]. Patients receiving matched therapies who had fewer prior therapies showed better PFS and OS than those who had received multiple prior therapies [46, 59].

### 3.8 Genomic Patterns Within Tumour Samples

Generally, median TMB in adult cancers (with known origin site) is lower than in CUP (4 versus 14 mutations per megabase [MB], respectively) [40]. High TMB is seen in 10%–25% of individuals with aggressive, advanced and hard‐to‐treat cancers [52, 87]. Within a cohort receiving immunotherapy, high TMB (≥ 20 mutations/MB) had a better response rate (58% versus 20%) and longer median PFS (12.8 versus 3.3 months) than low TMB (1–5 mutations/MB) or intermediate TMB (6–19 mutations/MB) [114].

High HRD scores were observed in 75% (*n* = 347/501) of a cohort with advanced solid tumour malignancies, including gastrointestinal, genitourinary and other rare cancers [115]. High MSI was identified in 2%–3% of samples from general cancer cohorts [87, 116, 117]. In one study, Lynch syndrome was diagnosed in 16% of individuals with high MSI and 2% of those with intermediate MSI, only half of whom presented with colorectal cancer or endometrial cancer [116]. A separate study used methylation to refine diagnoses, hypermethylation as a biomarker of response to therapy and aberrant methylation as a target for matched therapies [67].

### 3.9 Circulating Tumour DNA (ctDNA)

A liquid biopsy is minimally invasive and can be collected at multiple timepoints [15]. In early‐stage cancers, the frequency of ctDNA is low (< 1% of cell‐free DNA [cfDNA]) [118], but can exceed 70% in metastatic disease [119]. Tumour ctDNA is positively correlated with tumour stage, inversely related to patient age, higher in those assigned male at birth and varies according to tumour type [120]. Up to 99% of ctDNA samples meet the required quality standards for analysis [121, 122]. Sensitivity in detecting SNVs, indels and fusions from ctDNA is high (86%) [122, 123], and further improved by higher DNA input [124]. Additional P/LPVs can be detected in ctDNA compared to tissue biopsy due to the ability to capture greater tumour heterogeneity [123]. Concordance between tissue and ctDNA is 61%–85% [121, 122, 125, 126, 127, 128, 129, 130, 131]. Sensitivity can be compromised by physiological factors (e.g. obesity is negatively associated with ctDNA detectability) [132] or therapeutic interventions (e.g. surgical resection or commencing chemotherapy can cause a decline in ctDNA, regardless of clinical response) [133]. As ctDNA fragments are short (145–165 base pairs) [134], there is also reduced sensitivity (18.6%) for CNVs, large rearrangements and fusions [123]. Some cancer types shed less ctDNA for unknown reasons [129], and sensitivity can be assay dependent [135]. Logistically, a sufficiently large blood sample (10–20 mL) is required to yield enough ctDNA for analysis [15, 136], and there are specific blood processing and storage steps needed to maximise yields and mitigate the risk of ctDNA degradation [137]. Other challenges to applying ctDNA in practice include the need for high sensitivity assays (as the ctDNA is often only a small fraction of cfDNA) often requiring a greater depth of sequencing coverage (with higher costs), specialised bioinformatics analysis, longer turnaround time and reduced sensitivity relative to direct sequencing of tumour DNA [15, 122, 123, 138]. Finally, most services need to choose between tumour‐informed assays (higher sensitivity and specificity monitoring MRD) and tumour‐agnostic approaches (broader application across cancer cohorts but lower sensitivity) [16].

ctDNA can be used to monitor residual disease and treatment response through quantitative and qualitative assessment. Quantitatively, ctDNA levels are prognostic biomarkers whereby patients with advanced solid tumours with undetectable ctDNA have a longer median OS (68.4 months) than patients with detectable ctDNA (15.6 months) [129]. Furthermore, patients with lower levels of ctDNA have longer survival compared to patients with higher ctDNA levels [119, 132, 139]. Following cancer therapies, the presence of ctDNA is evidence of minimal residual disease and is associated with lower disease‐free survival and OS [65, 66]. Qualitatively, > 1 actionable variant in ctDNA is associated with poorer OS [119]. ctDNA analysis can detect treatment resistance variants [140] and MSI [141, 142]. Serial sampling can detect cancer recurrences after prolonged periods of remission [143]. Similarly, a recent study demonstrated high correlation between TMB estimates from tissue and blood ctDNA samples [142, 144, 145]. Furthermore, TMB from blood ctDNA was directly shown to predict response to immunotherapy in individuals with NSCLC [146].

Actionable ctDNA variants are identified more often in advanced/metastatic cancers (72%–85%) [119, 120, 122, 123, 130, 147, 148, 149], than in primary cancers (36%–62%) [121, 122, 125, 147, 149]. Cancer type affects the yield (e.g. 51% for glioblastoma, 86%–93% for NSCLC) [120, 125, 126], and larger gene panels are positively associated with the likelihood of detecting an actionable variant (e.g. 70–120 gene panels identify an actionable variant in 87%–91%) [121, 125, 126]. Eighty per cent of CUP patients (*n* = 442) had ≥ 1 P/LPV detected on ctDNA, of which 88% had distinct genomic profiles and 99.7% had potentially actionable variants [150]. The most frequently mutated genes across all metastatic cancers were *TP53* (38%–58%) [121, 125, 130, 148] and *EGFR* (11%–49%) [125, 149], while *EGFR* (26%–28%), *MET* (4%–6%), *KRAS* (4%) and *BRAF* (3%) were most commonly identified in primary cancers [126, 131, 149]. Sequencing ctDNA significantly increased the proportion of patients eligible for matched therapy compared to tumour testing alone (7.9% versus 6% for Tier I or II variants) [128]. The primary reason for not utilising personalised therapies based on ctDNA results was deteriorating patient condition [147]. Individuals with fewer prior therapies (< 4) experienced greater OS [119].

While ctDNA is typically extracted from whole blood, where central nervous system (CNS) involvement is suspected, ctDNA can be successfully extracted and sequenced from cerebrospinal fluid in 72% (*n =* 106/148) of cases [124]. In a cohort of 640 patients, blood ctDNA levels were lower in brain malignancies (< 50%) as compared to non‐CNS tumours (> 75%), which suggests that the blood–brain barrier may inhibit ctDNA circulation [118]. Concordance with previously sequenced tumour samples is high and ctDNA identified therapy‐related resistance variants in 15% (*n* = 11/75) of patients, and 3% (*n* = 2/75) had variants that independently diagnosed a new primary [124].

### 3.10 Testing for ctDNA in Asymptomatic Populations

Research is increasingly exploring the potential viability of using ctDNA as a biomarker for early cancer detection, possibly testing for many cancers at once (referred to as multi‐cancer early detection tests). ctDNA analysis is relatively non‐invasive and offers the possibility of early detection, which could improve prognosis [151]. However, screening ctDNA for common cancer P/LPVs presents a false‐positive risk as some variants are found in individuals with non‐malignant conditions (e.g. clonal haematopoiesis) [152]. Actionable variants may also be present but not detected, due to the early stage of the malignancy, the variant being present at a low level in the malignancy and/or the test not targeting the variant [15]. Even for ctDNA detection tests using more complex algorithms (e.g. aberrant methylation), sensitivity at an early stage is typically low (e.g. 27.5%) [153]. Finally, if a suspicious result is detected, further clinical testing may be required to detect or rule out cancer, as the person is asymptomatic and the primary site is unknown [15]. One study claimed that the false‐positive rate for the GRAIL‐Galleri ctDNA cancer screening was 0.5% [154]. When screening a large portion of the population (> 50 years of age), that could equate to hundreds of thousands of individuals without cancer undergoing extensive evaluation, which could place a substantial burden on health care systems and lead to adverse psychological and physiological effects for individuals [154].

## 4 Discussion

This narrative review consolidated evidence from 95 international original research publications focusing on the yield and clinical utility of germline and somatic P/LPVs, along with ctDNA testing, amongst adult cancer cohorts. We found that ~10% of adults with cancer and 13%–18% with rare cancers are found to carry germline P/LPVs [36, 37, 38]. Interestingly, this figure is equivalent to the a priori risk commonly required to offer germline testing based on family and medical history [7, 155]. The frequencies of germline P/LPVs are lower in adults than in children, adolescents and young adults (16%–18%) [62, 63, 156]. Rarer cancers in young individuals also have a much higher germline P/LPVs frequency (e.g. 69% in adrenocortical carcinoma tumours and 40% in retinoblastoma) [63, 157, 158, 159, 160] than rare cancers in adults (e.g. 25%–29% in gastrointestinal stromal tumours and 23% in phaeochromocytomas/paragangliomas) [34, 39, 54, 72]. Certain methodology variations are positively associated with higher detection rates of germline P/LPVs, including the number of genes sequenced [37, 39, 161], inclusion of moderate penetrance genes (18% yield) [36], sequencing matched tumour‐normal pairs [162, 163], and recency of study [13, 34, 38, 39, 41, 54]. The increased variant detection rate over the past 5 years is partially attributable to the expanded number of P/LPVs in ClinVar, the leading international database for germline variant classification [164]. Of note, not all populations are well represented in clinical databases, which compromises the sensitivity of identifying and actioning genomic cancer variants in diverse ancestral groups [165, 166].

Over half of germline P/LPV carriers are offered genotype‐directed therapies [39, 54], which can increase PFS and OS [84]. Germline variants can reveal contraindicated treatments [167], and germline pharmacogenomic testing can predict adverse responses to specific chemotherapies [168]. Germline P/LPVs can inform future malignancy risk for patients and unaffected family members, which guides screening and/or prophylaxis, leading to better clinical outcomes [26, 27, 28, 29] and potential health economic benefits [169, 170]. Given these multiple possible positive impacts, it is worth emphasising that half of germline P/LPV carriers identified in cancer genomic profiling would not have satisfied the existing eligibility criteria for germline testing [36, 73], and almost two‐thirds of germline carriers presented with cancers lacking explicit hereditary cancer testing guidelines [35]. Similarly, up to 70% of asymptomatic *BRCA1/2* P/LPV carriers in biobanks reported a negative family history [82]. These findings suggest that broader genomic testing criteria could maximise sensitivity and democratise access to clinical benefits for individuals and families. However, widespread testing may also identify individuals at inherently lower risk. The United Kingdom Biobank study analysis found that the average cancer risk appeared to be lower in germline carriers in the biobank as compared to the *BRCA1/2* carriers with a positive family history, suggesting that the nature of the variant and/or the genetic background modifies risk [82]. As variant curation and gene–gene interactions continue to improve, broader testing criteria are likely to detect a greater proportion of individuals at increased risk and customise management accordingly.

Typically, 73%–80% of individuals with cancer harbour a somatic P/LPV which informs diagnosis, prognosis and/or treatment [13, 38, 40, 41, 42, 43, 44, 45, 46, 47, 48, 49, 50]. The rate of detection was associated with the primary cancer site, disease stage and comprehensiveness of genomic sequencing. Somatic P/LPVs informed or refined diagnosis in a minority of patients with advanced disease (4%–10%) [13, 53], but up to half of patients with CUP [13]. Between one third and one half of individuals with cancer are recommended a treatment based on their molecular profile [13, 45, 46, 47, 53, 55], of which 33%–45% receive it [13, 38, 40, 41, 43, 44, 45, 46, 47, 48, 49, 50, 52, 59, 87, 99, 100, 101, 104]. Most patients accessed matched therapies through clinical trials. The main reasons for not accessing tailored treatments in clinical and non‐clinical trial settings included deteriorating patient condition, patient preference, limited access to targeted treatment and current or prior use of matched therapy. Multiple studies demonstrated that matched therapies were associated with better response rates or control rates [43, 44, 46, 49], longer median PFS [44, 49, 51], greater median OS [44, 49] and improved OS [56] as compared to individuals receiving standard of care or unmatched therapies. Furthermore, longer PFS and OS were observed in individuals receiving Tier I or II therapies as compared to treatments with lower levels of evidence [49, 56, 58, 59, 101]. The availability of matched, precision therapies is likely to continue to expand in the foreseeable future [171]. Importantly, matched therapies are considerably more expensive [172], less accessible [173] and can cause considerable adverse effects [174].

Within the past 5 years, there has been an increasing focus on the potential for ctDNA to augment cancer genomic testing, given improved feasibility and sensitivity [121, 122, 123, 175]. Furthermore, ctDNA can identify additional SNVs not detected on biopsy sequencing, but the larger variant detection is compromised by the short ctDNA fragment length [123]. Variant detection in ctDNA varies depending on physiological and clinical factors, most notably cancer type and stage [119, 120] and there are logistical challenges to the collection and timely processing of samples [15, 137, 138]. Diagnostically, the greatest promise of ctDNA lies in molecular profiling of individuals with inaccessible cancers or those of unknown primary. CUP accounts for 3%–5% of all cancers, are aggressive, difficult to treat and can usually only be profiled through biopsy of metastases [176]. However, ctDNA analysis detected actionable variants in most studied cases [150]. Prognostically, overall ctDNA levels can be a biomarker for response to treatment and/or residual disease and predict median and OS [65, 66, 129, 132]. Actionable variants are more frequently detected in ctDNA in metastatic cancers than primary cancers [122, 149], but utilising ctDNA significantly increases the identification of matched therapy options [128]. There is increasing interest in using patterns of variants in ctDNA (e.g. TMB and MSI) to detect recurrences and predict treatment response [141, 142, 143, 144, 145, 146]. Although ctDNA is not yet a practical screening tool for the healthy general population, such screening could possibly benefit individuals with germline P/LPVs in hereditary cancer predisposition syndrome genes, particularly in cancers for which there is no agreed upon screening modality or as an adjunct to other biomarkers. Both consumers and healthcare providers are enthusiastic about the potential value of ctDNA for screening, early detection and reduced imaging in this high‐risk population [177, 178], but longitudinal studies are needed to determine the feasibility, acceptability and long‐term effectiveness.

Despite the benefits of cancer genomic testing, it is not universally used in clinical practice. The European Society of Medical Oncology administered surveys to evaluate the utilisation of cancer genomic testing in 48 European countries and found that it was not accessible in many countries due to the cost and availability of tests and corresponding treatments [173]. Within individual countries, inequities of access are likely to further disadvantage marginalised groups. Of note, the greater response in individuals with fewer prior therapies [46, 59, 103] and poor utilisation of matched treatments due to deteriorating patient condition [110], makes a case for further research to establish where cancer genomic profiling should be used as a frontline test. Frontline testing could facilitate the selection of targeted therapies from the outset, avoid exposure to ineffective therapies and side effects, minimise the accumulation of treatment‐resistant variants, monitor disease progression and treatment response over time and improve response rates and outcomes.

## 5 Strengths and Limitations

This review captured larger international studies from the past 7 years that investigated the germline and/or somatic profile of diverse adult cancer types. Most selected studies originated in the United States, while some countries had a single large study reported, which limits generalisability to other populations and population subgroups. Most papers reported on research studies, as opposed to clinical practice and intentionally recruited patients with relapsed, resistant or metastatic disease. Fewer recruited all patients with cancer, especially those at initial presentation. A common limitation in precision medicine studies is the lack of prospective randomisation and appropriate controls. Additionally, few studies performed paired‐sample analyses to capture variability in variants within individuals/tumours and corresponding response to treatment over time. All these variabilities limit the ability to conduct a meta‐analysis. Our narrative review approach means that it is unlikely that we captured all eligible studies, while our exclusion of individual cancer studies limited the comprehensiveness of our analysis, particularly as it pertained to the sensitivity and specificity of ctDNA analyses, which can vary depending on cancer type.

## 6 Conclusion

Cancer genomic profiling can identify germline and somatic variants, which can inform prevention, diagnosis, prognosis and treatment. The impact on CUP in all three aspects of care is particularly significant. While most studies focused on advanced disease, patients undergoing genomic profiling immediately following diagnosis also benefited from molecular profiling. Response and survival data papers show that individuals receiving matched therapies have improved outcomes as compared to standard of care or those receiving unmatched therapies. Furthermore, those exposed to fewer prior therapies showed a greater response than those with multiple prior therapies. ctDNA profiling will likely play an increasingly important role in cancer care in the foreseeable future, though further studies are needed regarding the feasibility and acceptability of widespread implementation and the associated health economic analyses.

## Author Contributions

**Emily DeBortoli:** conceptualization (supporting), formal analysis (equal), methodology (equal), writing – original draft (equal), writing – review and editing (equal). **Ella McGahan:** formal analysis (supporting), methodology (supporting), writing – original draft (supporting), writing – review and editing (supporting). **Tatiane Yanes:** formal analysis (supporting), supervision (supporting), writing – review and editing (supporting). **Jennifer Berkman:** writing – review and editing (equal). **Lauren G. Aoude:** data curation (equal), writing – review and editing (equal). **Amelia K. Smit:** writing – original draft (equal), writing – review and editing (equal). **Akira Gokoolparsadh:** writing – review and editing (equal). **Azure Hermes:** writing – original draft (equal), writing – review and editing (equal). **Lyndsay Newett:** writing – review and editing (equal). **Mackenzie Bourke:** writing – review and editing (equal). **Susan Hanson:** conceptualization (equal), writing – review and editing (equal). **Helen Hughes:** conceptualization (equal), writing – review and editing (equal). **Oliver Hofmann:** writing – review and editing (equal). **Ilias Goranitis:** writing – review and editing (equal). **Rebekah McWhirter:** writing – review and editing (equal). **Vivienne Milch:** conceptualization (equal), writing – review and editing (equal). **Julia Steinberg:** writing – original draft (equal). **Aideen McInerney‐Leo:** conceptualization (equal), formal analysis (equal), methodology (equal), writing – original draft (equal), writing – review and editing (equal).

## Ethics Statement

This is a narrative review and therefore no ethical approval is required.

## Consent

The authors have nothing to report.

## Conflicts of Interest

The authors declare no conflicts of interest.

## Supporting information


**Table S1.** Search terms.

## Data Availability

All data relevant to the study are included in the article or uploaded as Supporting Information S1.
