# Supplementary material for: Utility of Germline, Somatic and ctDNA Testing in Adults With Cancer
Source: Cancer Med. 2025 Aug 1;14(15):e71080. doi: 10.1002/cam4.71080 (PMC12314551; doi:10.1002/cam4.71080)
Supplement: Supplementary file 1 — Table S1. Search terms. [file CAM4-14-e71080-s001.docx]

**Supplementary table 1**: search terms

| **Search area** | **Search terms** |
| --- | --- |
| Context | Cancer OR Oncology OR Carcinoma OR Tumour OR Neoplasm OR Malignancy |
| Field area | Genomic OR Genetic OR Germline OR Somatic OR Hereditary OR Mutation OR Alteration OR Variant OR Pathogenic OR Molecular OR Circulating tumour DNA (ctDNA) |
| Subject specific | Sequencing OR Profiling OR Liquid biopsy OR Whole-exome sequencing (WES) OR Whole Genome Sequencing (WGS) OR RNA-sequencing OR Next-generation sequencing (NGS) OR Personalised OR Precision OR Targetable OR Actionable OR Comprehensive OR Diagnosis OR Prognosis OR Therapeutic OR Clinical utility OR Tumour mutation burden (TMB) OR Homologous repair defect (HRD) OR Microsatellite instability (MSI) OR Aberrant methylation OR methylation |
